# Serum proteomic networks associate with pre-clinical rheumatoid arthritis autoantibodies and longitudinal outcomes

**DOI:** 10.3389/fimmu.2022.958145

**Published:** 2022-09-08

**Authors:** Liam J. O’Neil, Xiaobo Meng, Caitlin Mcfadyen, Marvin J. Fritzler, Hani S. El-Gabalawy

**Affiliations:** ^1^ Department of Internal Medicine, Manitoba Centre for Proteomics and Systems Biology, University of Manitoba, Winnipeg, Canada; ^2^ Department of Internal Medicine, University of Calgary, Calgary, Canada

**Keywords:** rheumatoid arthritis, proteomics, pre-clinical, jak-stat, prevention, machine learning

## Abstract

**Objectives:**

The development of autoantibody directed towards citrullinated proteins (ACPA) are predictive of RA in at-risk individuals. The biological events that underpin loss of immune tolerance and progression into inflammatory arthritis are not known. We sought to identify serum proteomic alterations that drive autoantibody formation, persistence and progression into inflammatory arthritis in a cohort of first-degree relatives (FDR) of RA patients.

**Methods:**

We studied baseline serum samples from a cohort of Indigenous FDR (n = 147) and quantified serum proteins using a 48-plex platform. Longitudinal outcomes were defined on the basis of ACPA status and progression into inflammatory arthritis (IA). K-means clustering, differential expression, and principal components analyze group differences. A co-expression module analysis was used to identify enriched networks. Random forest was used to classify ACPA positive samples, while network analysis was used to understand underlying biological processes based on protein expression.

**Results:**

We defined 6 proteomic clusters, with enrichment of ACPA positive samples in one of the clusters. 23 of 24 differentially expressed proteins in ACPA positive samples were upregulated. A co-expression network was enriched in ACPA positive sera and individuals who progressed into IA. Random Forest achieved an area under the curve of 0.767 to classify ACPA positive sera in a test dataset. Network analysis revealed upregulation of JAK-STAT signalling as being activated in those at highest risk to develop future IA.

**Conclusions:**

The serum proteome provides a rich dataset to understand biological processes in ACPA seropositive individuals. A combination of serum biomarkers, including ACPA, may predict future arthritis onset in at-risk individuals.

## Key Messages

- Serum proteomics classifies ACPA positive from ACPA negative first-degree relatives
- Protein co-expression network associates with ACPA status and progression into inflammatory arthritis
- JAK-STAT signalling is upregulated in those at highest risk to develop inflammatory arthritis

## Introduction

Rheumatoid Arthritis (RA) is a common, systemic autoimmune disease that primarily targets the synovial joints (1). Without immunosuppressive treatment, RA leads to chronic pain and joint damage which ultimately impacts on functional status and quality of life (2). Despite decades of research, the etiology of RA remains elusive. The study of pre-clinical RA, the stage prior to the onset of clinically detectable joint inflammation, has provided key insights into how a state of subclinical systemic autoimmunity transitions to an immune mediated inflammatory arthritis (3, 4). The hallmark of this pre-clinical RA period is the detection of specific autoantibodies, particularly those directed towards citrullinated peptide epitopes (anti-citrullinated protein antibodies; ACPA), along with autoantibodies directed towards other post-translational modifications (3, 5, 6). These autoantibodies, most of which ultimately persist throughout the course of this chronic disease, can be detected months and even years prior to disease development (7).

Despite these insights, the complex biological events that initiate, propagate, sustain, and amplify pre-clinical RA autoimmunity remain poorly understood. It has now been clearly shown by multiple groups that ACPA seropositivity in an otherwise unaffected individual is a strong risk factor for future RA development (5, 8, 9). Importantly, we have also shown in a prospective longitudinal cohort of at-risk, but unaffected first-degree relatives (FDR) of Indigenous North Americans (INA) that only a proportion of individuals in whom ACPA are detected at any specific timepoint will actually go on to develop RA, and indeed many seropositive individuals become seronegative over time (5). These observations suggest that subsequent to an initial break in immune tolerance characterized by the development of ACPA, and possibly other anti-modified protein antibodies (AMPA), there are regulatory mechanisms that may serve to control these potentially harmful processes, and that these mechanisms are effective in preventing the progression towards pathogenic autoimmunity in a substantial proportion of individuals. Indeed, it can be hypothesized that the development of this autoimmune disease potentially represents a failure of these regulatory mechanisms.

Networks of cytokines that regulate broad inflammatory processes are implicated in the pathogenesis of RA (10). Numerous studies have demonstrated the upregulation of these networks in both blood and the synovium of RA patients (11). These proteins promote several key aspects of RA pathogenesis, including the formation of autoantibodies, sustaining inflammatory arthritis and promoting joint damage. Indeed, cytokine networks are implicated in the function of lymphocytes (both T (12) and B (13) cells), innate immune cells (macrophages (14), neutrophils) and stromal cells such as fibroblast-like synoviocytes (15), responsible for local joint invasion and destruction of cartilage. Importantly, targeting these specific cytokine networks has led to the development of therapeutics that have altered patient outcomes drastically (16). However, precisely when these cytokine networks initiate, and how they contribute to pre-clinical RA pathogenesis is not known. Defining functional modules (17) and disease-specific cytokine hierarchies across all stages of RA will help provide key insights into therapeutic targets, disease pathogenesis and personalized medicine approaches.

Our prospective longitudinal cohort study of the FDR of INA RA patients has afforded us the opportunity to explore the pre-clinical period of individuals who ultimately developed seropositive RA, comparing them to individuals in whom autoantibodies were not detected, and particularly to ACPA seropositive individuals in whom the autoimmune processes did not appear to progress during the observation period (5). As such, we have characterized the ACPA themselves and shown that epitope spreading (5), and a high degree of variable region glycosylation (18) were both associated with clinical disease development. Moreover, we used exploratory broad-based proteomic approaches to detect proteomic signatures that predicted RA development with a high degree of accuracy (19). These signatures were enriched for proteins associated with innate immune mechanisms. In the current study we present data regarding the circulating cytokine/chemokine profiles of these groups of individuals, and how these profiles relate both to the RA autoantibodies, and to the ultimate development of RA.

## Methods

### Cohort overview and sample selection

Methods and protocols for patient recruitment for this study were described in our group’s previous work (20). In brief, INA RA probands who met the 2010 ACR/EULAR criteria (21) were approached to help recruit their eligible FDR for longitudinal follow up. We expected that a proportion of these patients would go on to develop inflammatory arthritis. Both RA probands and FDRs were required to have at least 3 grandparents with INA ethnicity by self-report. Participants had to be 18 years of age or older. At baseline, all FDR were examined by a rheumatologist to confirm the absence of clinical synovitis. Participants then entered the study and underwent annual examination for the presence of clinical synovitis. Between the annual evaluations, FDRs were instructed to report any new symptoms suggestive of arthritis. Clinical assessment by a member of the research team took place as soon as possible, to assess reported symptoms. If synovitis was unequivocally detected in one or more joints by a rheumatologist, the individual was deemed as having “progressed” to having inflammatory arthritis. Serum samples were collected at all study visits and stored at -20°C for future studies. Anti-CCP antibodies were detected using CCP3 ELISA (Inova), using manufacturer’s cut-off values to determine positivity. We assigned longitudinal outcomes in ACPA positive samples as: 1. Seroconversion (ACPA negative after follow-up) 2. ACPA positive persistence or 3. ACPA positive with progression into inflammatory arthritis. These pre-clinical baseline states were chosen on the basis of increasing risk for future development of seropositive RA.

### Ethics

All study participants provided informed consent in accordance with the Declaration of Helsinki. The Biomedical Research Ethics Board of the University of Manitoba approved all aspects of the study (Board approval number HS14453). Specific community research agreements were put in place with the study communities. Consistent with the guidelines from the Canadian Institutes of Health Research for conducting research involving indigenous people in Canada, we established an arthritis advisory board, to provide indigenous oversight for the study.

### Serum proteomics

Serum proteins were analyzed using a Luminex xMAP 48-plex cytokine/chemokine/growth factor panel (Millipore, HCYTA-60K) which included: IL1A, CXCL1, IFNA2, IFNG, IL1B, CCL3, CCL4, TGFA, TNF, LTA, CXCL9, IL25, PDGFB, FGF2, FLT3LG, CSF3, IL1RN, IL2, IL4, IL5, IL6, IL7, IL8, IL9, IL10, IL12B, IL13, IL15, IL17A, IL17F, IL27, CCL2, CCL7, MCSF, PDGFA, VEGFA, IL12A, CXCL10, IL18, IL22, CCL5, CSF2, IL3, MDC, IL25, CX3CL1, EGF and CD40LG. Standard curves for all analytes were generated using 5-parameter logistic regression on 8 standard samples. Individual analyte sensitivity values are available in the MILLIPLEX^®^ MAP protocol provided with the manufacturer’s kit. Samples were analyzed using a Luminex 200 luminometer and the concentration of analytes was reported in pg/mL. Gene names were used to annotate proteins for readability. Protein expression was log-transformed to normalize, and assessed for distribution/quality control (**Figure S1**) and proteins (CCL5, CSF2, IL3) were removed if > 25% of the samples fell outside of the assay range.

### ACPA N-linked variable domain glycan

Methods to extract ACPA and quantify glycan composition of the variable domain have been published previously (22). In brief, ACPA and non-ACPA IgG are captured and eluted. Glycans are released, purified and profiled using ultra-high performance liquid chromatography (UHPLC) and custom software. The Fab glycosylation is expressed as a % of Fc glycans for each IgG sample. Data for Fab glycosylation of ACPA was available for 17 samples in this study.

### Data analysis

All data was analyzed in the R (v4.0.2) environment using RStudio (2022.02.0 Build 443). Dichotomous group differences were assessed using chi-square test, while continuous outcomes were analyzed using Wilcoxon rank sum test for non-parametric assessments, and t-test for parametric assessments. Dunn’s was used to evaluate non-parametric differences between multiple groups, adjusted for multiple comparisons (Holm). Correlation between two continuous variables was evaluated using spearman rank correlation. A correlation matrix of protein expression was created using Pearson correlation, adjusted for multiple comparisons (Bonferroni). Principle components analysis was undertaken using multi-dimensional scaling (MDS). Consensus clustering (*ConsensusClusterPlus)* was used to identify sample clusters using k-means clustering, anchored to cluster optimization based on the area under the cumulative distribution function (CDF) curve. The clinical data collected that was used for the analysis was complete and without any missing values.

Differentially expressed proteins (DEPs) were determined using pairwise t-test with statistical adjustment for multiple comparisons (Benjamini-Hochberg). Proteins considered significant required a fold change of greater than 2 or less than 0.5, along with an adjusted p-value of < 0.05. Weighted gene correlation network analysis [*WGCNA* (23)] was used to identify protein modules based on hierarchical clustering (23). The following parameters were used to define modules: power = 10, deepSplit = 2, maxCutHeight = 0.5. The module score was created by extracting the scaled protein expression data, multiplying each protein by the correlation value (to weight towards strong module members) and summing each protein.

Network analysis was undertaken using *clusterprofiler*(24) and enriched for gene ontology, while drug/disease data was extracted from EnrichR’s web tool (25). Drug data was annotated manually. In brief, drug networks containing at least 6 protein module members with a p-value of < 4.0 x 10^-5^ were selected then classified based on literature review. Visualizations were undertaken using the packages *ggplot2*(26), *corrplot*(27)= and *pheatmap*(28)*.* A random forest classification model was trained on 75% of the cohort samples, with the intent of classifying ACPA positivity samples from ACPA negative, using the *tidymodels*(29) package in R (trees = 1000). Variable importance was extracted from the model using the package *vip*(30).

## Results

### Upregulation of soluble mediators in ACPA positive compared to ACPA negative FDR

**Figure 1:**
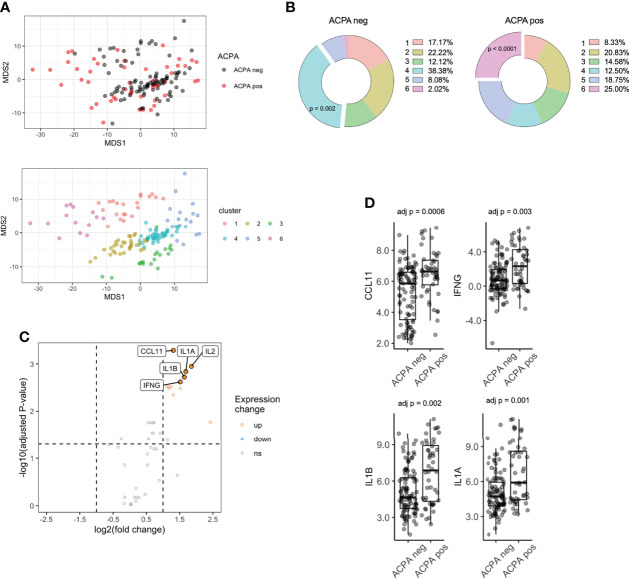
Serum proteomics differences between ACPA positive and ACPA negative FDR of RA patients. **(A)** Multi-dimensional scaling (MDS) plot of FDR sera based on 48-plex proteomics coloured by ACPA status (top) or consensus cluster (bottom, n = 6). **(B)** Cluster assignment enrichment based on ACPA status. Clusters analyzed by chi-square test. **(C)** Volcano plot of differentially expressed proteins, colored by upregulated (fold change > 2) and downregulated (fold change < 0.5) in ACPA positive group. Analyzed by pairwise t-test adjusted by Benjamini-Hochberg. **(D)** Box plots of CCL11, IFNG, IL1B, IL1A comparing ACPA positive and ACPA negative groups. ACPA, anti-citrullinated protein antibodies; FDR, first-degree relatives; CCL11, C-C Motif Chemokine Ligand 11; IFNG, Interferon gamma; IL1B, Interleukin 1 Beta; IL1A, Interleukin 1 Alpha. ns = not significant.

Using a 48-plex cytokine/chemokine array as outlined in the Methods, we initially sought to determine whether there were group differences in the circulating profile of ACPA positive FDRs (n = 48) compared to ACPA negative FDRs (n = 99), irrespective of the outcome in the ACPA positive group. We selected serum samples from 117 individuals (n = 147 serum samples) to balance age and sex, and to include participants with well-defined longitudinal outcomes (**Table 1**). Importantly, all sera analyzed were from the pre-clinical phase of the longitudinal cohort study and none of the participants had inflammatory arthritis. Principle components analysis (PCA) performed using all of the measured proteins revealed substantial overlap between ACPA negative and ACPA positive samples (**Figure 1A**).

**Table 1:** Demographics of serum samples.

	ACPA negative (n = 99)	ACPA positive (n = 48)	p-value
**% Female (n)**	68.7 (68)	77.1 (37)	0.389
**Age (IQR)**	43.8 (22.8)	35.9 (25.0)	0.239
**BMI (IQR)**	28 (8.9)	28.7 (8.9)	0.574
**CCP3 (IQR)**	2.6 (5.9)	141.0 (224)	p < 0.0001

BMI, body mass index. Analyzed by Wilcoxon rank sum test and chi-square.CCP3: third generation cyclic citrullinated protein antibodies.

K-means clustering identified 6 clusters (**Figure S2**) of samples, which separated on the basis of protein expression in PCA space (**Figure 1A**). Interestingly, cluster enrichment was observed on the basis of ACPA status, with Cluster 4 containing a significant proportion of ACPA negative samples (38.4% overall, p = 0.002, 86.3% within cluster) and Cluster 6 containing a significant proportion of ACPA positive samples (25.0% overall, p < 0.001, **Figure 1B**, 92.8% within cluster), and individuals who progressed into inflammatory arthritis (50.0% overall, 42.9% within cluster, p = 0.0003). To determine other factors that may influence circulating cytokines expression, we analyzed cluster assignment differences in Sex, Age and BMI (**Table 2**). Sex differences did not reach statistical significance; however, the proportion of Females in Cluster 6 (enriched in ACPA positive samples) was highest amongst the 6 clusters (92.8%). Age was the lowest in cluster 5 (27.9), which reached significance when compared to cluster 2 (46.4), cluster 3 (49.3) and cluster 4 (46.0). CCP3 level was highest in Cluster 6 and reach statistical significance in comparison to Cluster 1 and 4. BMI showed no significant differences based on cluster assignment.

**Table 2:** Demographics of serum samples split by cluster assignment.

	Cluster 1(n = 21)	Cluster 2(n = 32)	Cluster 3(n = 19)	Cluster 4(n = 44)	Cluster 5(n = 17)	Cluster 6(n = 14)	p-value
**% Female (n)**	71.4 (15)	81.3 (26)	68.4 (13)	68.2 (30)	47.1 (8)	92.8 (13)	p = 0.075
**Age (IQR)**	34.1 (24.4)	46.4 (18.4)	49.3 (21.6)	46.0 (25.2)	**27.9 (10.8)**	39.1 (23.6)	5 vs 2 (p = 0.002) 5 vs 3 (p = 0.01) 5 vs 4 (p = 0.008)
**BMI (IQR)**	31.1 (13.3)	28.0 (10.3)	27.6 (7.6)	29.1 (7.5)	27.4 (8.4)	27.3 (8.7)	ns
**% Progression (n)**	0 (0)	9.4 (3)	10.5 (2)	0 (0)	5.9 (1)	**42.9 (6)**	p = 0.0003
**CCP3 (IQR)**	2.6 (16.5)	5.2 (107.0)	6.5 (87.6)	4.45 (7.29)	66.8 (107)	**132.0 (252.0)**	6 vs 1 (p = 0.01), 6 vs 4 (p = 0.002)

CCP3, third generation cyclic citrullinated protein antibodies; BMI, body mass index; ACPA, anti-citrullinated protein antibodies; ACPA, ACPA negative; ACPA seroconv, ACPA seroconversion, ACPA +: ACPA positive, ACPA + prog: ACPA positive progressor. Analyzed by Dunn’s test and chi-square; corrected for multiple comparisons with Benjamini-Hochberg. Bold, means statistically significant vs multiple clusters. ns = not significant.

Next, differential expression of proteins (DEPs) was determined between ACPA positive and negative samples, which identified 13 DEPs, all of which were upregulated after adjusting for multiple comparisons (**Table S1** **;** **Figure 1C**). The highest upregulated proteins in ACPA positive samples included CCL11, IFNG, IL1B and IL1A (**Figure 1D**). These data suggest that differentially expressed soluble mediators in ACPA positive sera are nearly exclusively upregulated, suggestive of a pro-inflammatory state that is synchronous with subclinical RA autoimmunity.

### Longitudinal outcomes differ on the basis of cytokine profiles in FDR of RA patients

Given the DEPs identified between ACPA positive and negative individuals, we next sought to understand if the upregulation of cytokines/chemokines varied on the basis of longitudinal outcomes. As indicated above, we have previously observed that a proportion of individuals who are ACPA positive will seroconvert to an ACPA negative state over follow up (5). We classified longitudinal outcomes in ACPA positive samples as: 1. Seroconversion (ACPA negative after follow-up) 2. ACPA positive persistence or 3. ACPA positive progression into inflammatory arthritis (**Table S2**, see Methods). Prior to analyzing differences between these groups, we sought to determine the longitudinal stability of the levels of the analytes in samples from persistently ACPA negative individuals. This analysis revealed no statistically significant differences in any of the proteins after a mean of 782 days between sample collection, suggesting protein expression is stable over time in individuals whose autoantibody and clinical status did not change (**Figure S3** **;** **Table S3**).

**Figure 2:**
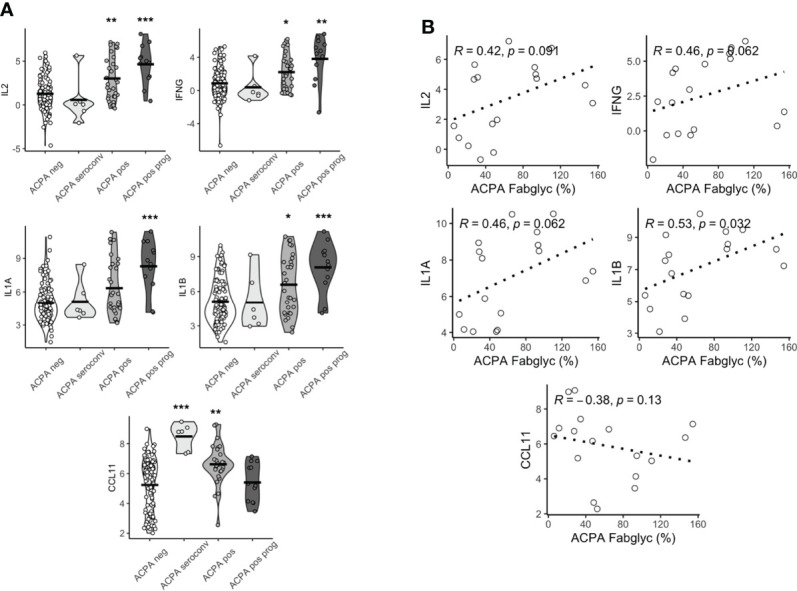
Baseline serum proteomics varies based on longitudinal outcomes. **(A)** Violin plots of CCL11, IFNG, IL2, IL1A, IL1B based on longitudinal outcomes, ACPA negative, ACPA seroconversion, ACPA positive and ACPA positive progression (prog). Analyzed by Dunn’s test adjusted by holm. ***p < 0.001, **p < 0.01, *p < 0.05. **(B)** Spearman correlation plots of IL2, IFNG, IL1A, IL1B and CCL11 and ACPA Fab glycosylation. ACPA, anti-citrullinated protein antibodies; Fab, variable domain region of IgG; CCL11, C-C Motif Chemokine Ligand 11; IFNG, Interferon gamma; IL1B, Interleukin 1 Beta; IL1A, Interleukin 1 Alpha; IL2, Interleukin 2.

We then compared the expression levels in the groups outlined above. We observed substantial differences in multiple proteins based on this clinical classification. IL2, IFNG, IL1A, IL1B all displayed a similar pattern of expression, where individuals who subsequently progressed to develop IA had the highest levels, while individuals whose seropositivity disappeared over time were no different compared to persistently ACPA negative controls (**Figure 2A** **;** **Supplementary Files**). Although this was observed for these pro-inflammatory cytokines, some proteins, for example CCL11, showed the opposite pattern, with the highest expression in individuals whose seropositivity resolved rather than the IA progressor group.

Since we (22), and others (18), had previously demonstrated that individuals in whom ACPA exhibited high levels of Fab glycosylation were most likely to progress to IA, we next sought to determine if there was a relationship between cytokine/chemokine expression patterns and Fab glycosylation levels of ACPA. We observed a significant positive correlation between the highest DEPs (IL2, IFNG, IL1A and IL1B) and Fab ACPA glycosylation (**Figure 2B**), which was only available for 17 samples. We also observed a negative but non-significant correlation between CCL11 and Fab ACPA glycosylation. This suggests that a specific serum cytokine/chemokine expression pattern is associated with heavily Fab glycosylated ACPA, a demonstrated major predictor of future RA.

### A co-ordinated serum protein network in pre-clinical RA is associated with arthritis onset.

**Figure 3:**
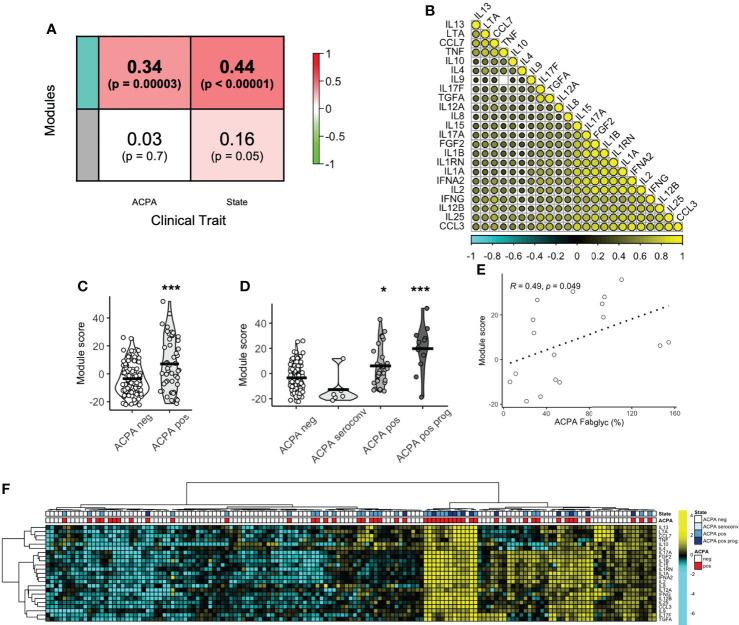
Protein co-expression network enriched in ACPA positive FDR. **(A)** Correlation coefficient between protein co-expression modules (Teal, Grey) and ACPA or longitudinal outcome (state), colored based on strength of association. **(B)** Correlation matrix of protein module members (Teal) colored based on strength of correlation (range -1 to 1). **(C)** Box plots of module score comparing ACPA positive and ACPA negative groups. Analyzed by t-test, *** p < 0.001. **(D)** Violin plots of module score based on longitudinal outcomes, ACPA negative, ACPA seroconversion, ACPA positive and ACPA positive progression (prog). Analyzed by Dunn’s test adjusted by holm. ***p < 0.001, *p < 0.05. **(E)** Spearman correlation plot of module score and ACPA Fab glycosylation. **(F)** Heatmap of co-expression network protein members ordered by hierarchical clustering and colored by ACPA status and longitudinal outcomes. ACPA, anti-citrullinated protein antibodies; Fab, variable domain region of IgG.

Given that several individual circulating cytokine/chemokine proteins were associated with a high risk of RA development, we next sought to define the co-ordination of the serum cytokine/chemokine proteome. Using a network algorithm (WGCNA), protein co-expression was split into 2 modules (Grey, n=22 and Teal, n = 23) on the basis of hierarchical clustering. The relationship between module eigengene expression score and predefined clinical traits was positive for the Teal module, with a significant correlation for both ACPA seropositivity (**Figure 3A**, R = 0.34, p < 0.0001) and longitudinal clinical outcomes (state; R = 0.44, p < 0.0001). Correlation between module members was indeed strong (range 0.27 to 0.92) as visualized in a correlation matrix (**Figure 3B**). A module score was calculated and found to be higher in ACPA positive FDR compared to ACPA negative FDR (7.15 vs -3.46, p = 0.0005, **Figure 3C**). When categorized into longitudinal clinical outcomes, compared to ACPA negative FDR, those who eventually developed arthritis (Progression) had the highest baseline module score (19.8 vs -3.46). Interestingly, there was no difference in serum module score between individuals who underwent ACPA seroconversion, and ACPA negative FDR. Numerically, modules scores increased based on clinical state, from ACPA negative (-3.46) and ACPA seroconversion (-12.8), to persistent ACPA positivity (6.06) and Progression into inflammatory arthritis (19.8, **Figure 3D**). Furthermore, module score correlated significantly with ACPA Fab glycosylation (**Figure 3E**, R = 0.49, p = 0.049). These data suggest that a co-ordinated protein network associates with key longitudinal outcomes in at-risk FDR of RA patients.

### Machine learning to classify ACPA positive from ACPA negative FDR

**Figure 4:**
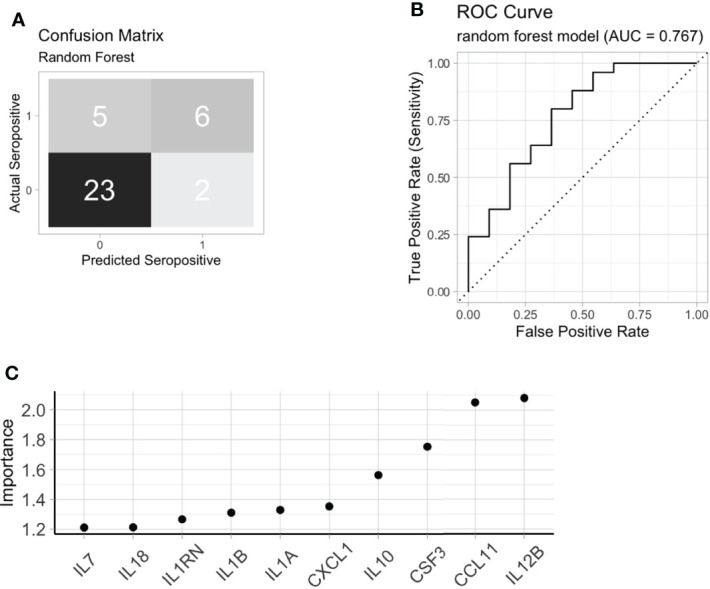
Machine learning to identify ACPA positive FDR. **(A)** A random forest model confusion matrix on a test cohort (n = 36). **(B)** Receiver operating curve (ROC) of random forest model to classify ACPA positive FDR from ACPA negative FDR based on test cohort. **(C)** Variable importance extracted from the random forest model. FDR: first-degree relatives, ACPA: anti-citrullinated protein antibodies.

ACPA seropositivity remains a key biomarker in predicting future RA. We were able to identify serum proteins that associated with ACPA status, however the utility of serum proteomics to classify pre-clinical autoantibody positive individuals is not clear. Using chi-square test, hierarchical clustering with DEP’s enriched in ACPA positive pre-clinical samples was able to classify ACPA positive individuals from ACPA negative (**Figure 3F**, p = 0.008). However, clustering performed poorly as a diagnostic test to identify ACPA positive samples even without the stringency of a train-test approach (Accuracy 58.5%, Sensitivity 72.9%, Specificity 51.5%). Thus, we next sought to determine the value of combining machine learning with serum proteomics to classify FDR with pre-clinical ACPA. Random forest (RF) classification achieved 100% accuracy in the training cohort (n = 104), and 80.5% accuracy in the test cohort (n = 36, Sensitivity 54.6%, Specificity 92.0%, **Figure 4A**). In the training cohort the RF model provided an area under the curve (AUC) value of 0.767 **(** **Figure 4B**). Model variable importance included identified predominant members of the protein module network that was identified previously **(** **Figure 4C**). These results suggest that ACPA status may be classifiable on the basis of serum proteomics, further strengthening the evidence that autoantibody positive individuals display a measurable co-ordinated serum protein expression profile that is distinct from FDR who are ACPA negative.

### Serum protein network proteins are linked to JAK-STAT activation and pharmaceutical targets

**Figure 5:**
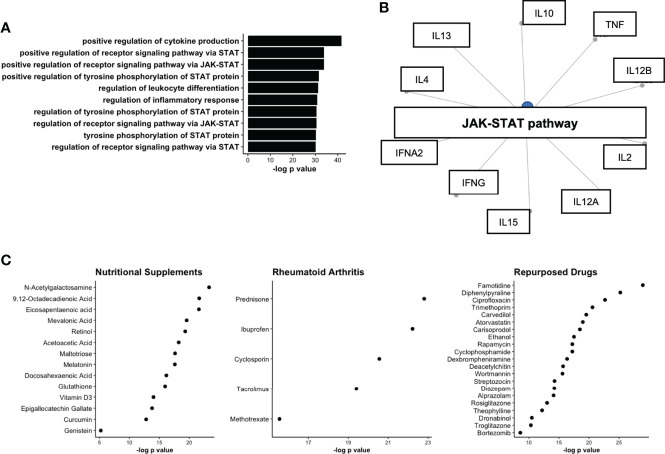
Network analysis of protein co-expression module upregulated in ACPA positive FDR. **(A)** Upregulated GO pathways based on proteins identified in co-expression network. **(B)** Gene concept plot displaying common node (JAK-STAT), connecting protein module members. **(C)** Drug network analysis split by broad classes of pharmaceutical including nutritional supplements, Rheumatoid Arthritis treatments, and drugs approved for other diseases/conditions (Repurposed). STAT, signal transducer and activator of transcription; JAK, Janus Kinase; ACPA, anti-citrullinated protein antibodies.

We next aimed to determine the underlying biological pathways of the module protein members, from the WGCNA analysis. Gene enrichment identified several pro-inflammatory pathways that were upregulated on the basis of module protein members. Notably, the pathway *positive regulation of cytokine production* contained 16 of 23 module members and was found to the most significantly enriched pathway (**Figure 5A**). The remaining top pathways all included references to JAK-STAT enrichment, a pathway known to be active in individuals with established RA (31). Indeed, *JAK-STAT activation* connected several module members, suggestive of a common node that might be responsible for the co-ordinated serum protein network (**Figure 5B**). We next sought to determine drug targets that could be deployed to disrupt this network and generate hypothetical treatment options for RA prevention studies. The Drug Signatures Database (*EnrichR*) identified 1030 drugs associated with expression of module proteins after adjustment (**Table S4**). Following strict filtering, 3 broad categories of drugs were identified 1. Nutritional Supplements 2. Drugs commonly used to treat RA (*Rheumatoid Arthritis*) and 3. Drugs that are commonly used in medical practise, but not typically for treatment of RA (*Repurposed*, **Figure 5C**). Notably, nutritional supplements identified included: Vitamin D3 and Omega-3 fatty acids, which have recently been shown to reduce incident autoimmune disease in a healthy population (32). These results suggest that serum protein networks upregulated in pre-clinical RA may be modulated with medications that target JAK-STAT signalling.

## Discussion

The biological processes that precede the onset of clinically detectable RA remain unclear despite extensive documentation by many groups after a prolonged pre-clinical period (5, 8, 33). This preclinical period is defined primarily by the presence of ACPA and other RA associated autoantibodies in otherwise unaffected individuals (33). Although the detection of ACPA remains a strong predictor of future RA development, we have previously demonstrated that a substantial proportion of prospectively followed ACPA positive FDR of RA patients do not develop RA, and indeed not uncommonly seroconvert to an ACPA negative state (5). This observation suggests that breaking immune tolerance to citrullinated endogenous autoantigens is not an irreversible process that inexorably leads to pathogenic autoimmunity and RA. As such, it of considerable interest to identify immunological process that not only associate with ACPA development, but also with the evolution and maturation of this autoimmunity to become pathogenic. In the current study we have focused on the circulating cytokine/chemokine networks that are cross-sectionally associated with ACPA seropositivity, and particularly on the utility of these networks in predicting future RA onset in individuals who ultimately developed RA. We demonstrate that these networks are enriched in proteins associated with activation of the JAK-STAT pathway, which is known to play a key role in RA pathogenesis.

Several studies of ACPA positive individuals who ultimately developed RA have shown that as RA onset approaches, the ACPA response exhibits epitope spreading with expansion of the targeted autoantigens and increasing levels of glycosylation in the variable region domains, both of which are associated with increases in the circulating levels of these autoantibodies (5, 18). These observations are consistent with an immunological maturation of the autoimmune response that requires close interactions between B and T lymphocytes, a process that typically takes place in germinal centers located in lymphoid tissues (1). It remains abundantly clear that sustainment and maturation of autoantibody production in pre-clinical RA is representative of a key step towards the onset of clinically apparent disease. Interrupting this process may be crucial in the prevention of RA in at-risk, clinically quiescent individuals (4).

We identified a co-expression network of serum proteins that associated with both baseline seropositivity status and longitudinal outcomes in this at-risk cohort. The network, made up of roughly 50% of our measured proteins, was enriched for biological for processes suggestive of JAK-STAT activation. It is well established that the JAK-STAT pathway plays a key role in orchestrating several important immune responses, many of which are upregulated in those with established RA (31). Perhaps the most convincing evidence of JAK-STAT activation in RA, is the robust efficacy of JAK inhibitors to control RA-related disease activity, achieving response rates that exceed those of TNF inhibitors, the gold standard of initial RA biologic therapy (34). The family of JAKS includes 4 tyrosine kinases including JAK1, JAK2, JAK3 and TYK2, which govern essential cytokine responses including IL-6, and type 1/2 interferons. Binding of a cytokine/chemokine to a respective receptor initiates an intracellular cascade of events, most importantly phosphorylation of JAK proteins, phosphorylation and dimerization of STAT, and translocation of STAT to the nucleus to initiate transcription of target genes (31). Despite its prominent role in RA, the role of JAK-STAT signalling in pre-clinical RA is not known, and our data is the first to associate JAK-STAT activation in pre-clinical autoantibody development and arthritis onset.

The interplay between autoantibody development and other dysregulated features of the immune system remains relatively unexplored, however we have previously observed innate immune protein signatures that associate with progression into RA (19). The present study builds off of this work, by seeking to address differences between FDR who are ACPA positive, compared to those that are not. Past studies have also indicated familial clustering of cytokines (35) and upregulation of type-1 interferon (36) in pre-clinical RA. It is entirely unclear if cytokines/chemokines drive autoantibody formation, or if ACPA themselves lead to dysregulated immune responses, which has been show in established RA (37, 38). Identifying activated biological pathways that facilitate autoantibody formation may provide an opportunity to deploy targeted therapies to modulate pre-clinical autoimmunity, with the intention of preventing future RA in seropositive individuals (39). Indeed, our data and others (36), suggests that key protein networks activated in pre-clinical RA such as JAK-STAT signalling, may be modulated by certain nutritional supplements, with the possible added benefit a favorable safety profile for deployment in prevention studies (32).

In conclusion, these data suggest that serum proteomics differ significantly on the basis of autoantibody status amongst FDR of RA patients, and a co-expression network of proteins associates with longitudinal outcomes in pre-clinical RA. Replication of these data in future studies will help establish a working model for predicting incident RA in at-risk populations using a combination of serum and clinical biomarkers.

## Data availability statement

The data presented in the study are deposited in the Mendeley Data Repository, doi: 10.17632/rf3vbm4ykm.1

## Ethics statement

The studies involving human participants were reviewed and approved by University of Manitoba REB. The patients/participants provided their written informed consent to participate in this study.

## Author contributions

LO’N conceptualized study, performed data analysis and wrote the manuscript. XM performed all CCP testing. CM maintains cohort, recruitment, clinical data. MF generated all of the proteomic data. HE=G conceptualized study and wrote the manuscript. All authors contributed to the article and approved the submitted version.

## Funding

The entirety of this work was funded by CIHR grant MOP77700.

## Conflict of interest

The authors declare that the research was conducted in the absence of any commercial or financial relationships that could be construed as a potential conflict of interest.

## Publisher’s note

All claims expressed in this article are solely those of the authors and do not necessarily represent those of their affiliated organizations, or those of the publisher, the editors and the reviewers. Any product that may be evaluated in this article, or claim that may be made by its manufacturer, is not guaranteed or endorsed by the publisher.
