# Supplementary material for: Serum proteomic networks associate with pre-clinical rheumatoid arthritis autoantibodies and longitudinal outcomes
Source: Front Immunol. 2022 Sep 8;13:958145. doi: 10.3389/fimmu.2022.958145 (PMC9492875; doi:10.3389/fimmu.2022.958145)
Supplement: Supplementary file 1 [file DataSheet_1.docx]

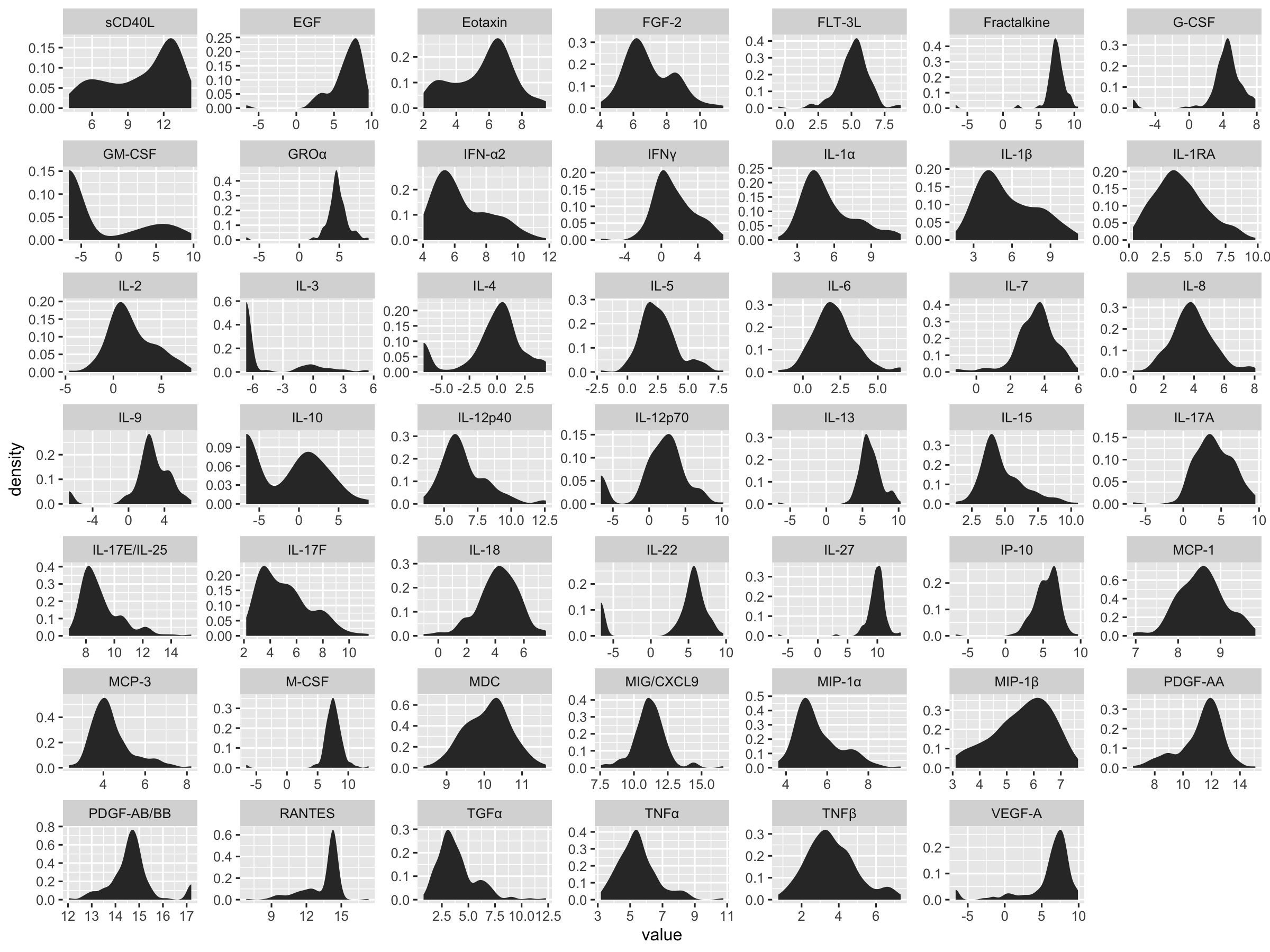


Figure S1: 48-plex serum protein data expression plotted followed log transformation.


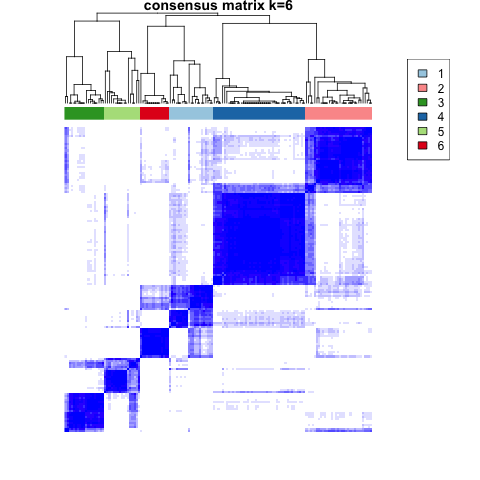


Figure S2: Consensus clusters (n = 6) based on 48-plex serum proteomic data.


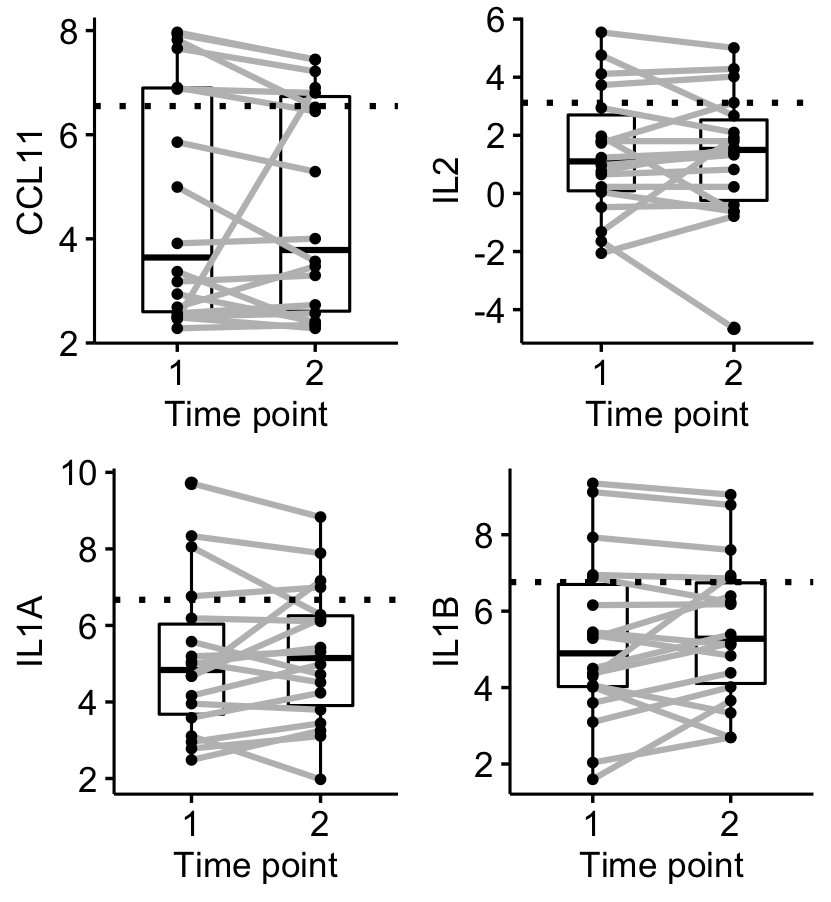


Figure S3: Paired expression between persistent ACPA negative samples. Mean time between samples 782 days. Dotted line represents the mean expression level for ACPA positive samples. ACPA: anti-citrullinated protein antibodies, CCL11: C-C Motif Chemokine Ligand 11, IL1B: Interleukin 1 Beta, IL1A: Interleukin 1 Alpha, IL2: Interleukin 2.

| protein | statistic | df | p.adj | p.adj.signif | fold_change | assignment |
| --- | --- | --- | --- | --- | --- | --- |
| CCL11 | -4.61 | 105.08 | 0.0005 | *** | 2.48 | up |
| IL2 | -4.31 | 71.66 | 0.0012 | ** | 3.61 | up |
| IL1A | -4.15 | 67.48 | 0.0015 | ** | 3.22 | up |
| IL1B | -3.97 | 72.01 | 0.0020 | ** | 3.12 | up |
| IFNG | -3.82 | 76.54 | 0.0025 | ** | 2.86 | up |
| IL12B | -3.62 | 60.44 | 0.0031 | ** | 2.37 | up |
| IL15 | -3.67 | 63.63 | 0.0031 | ** | 2.26 | up |
| IL25 | -3.70 | 58.16 | 0.0031 | ** | 2.17 | up |
| IFNA2 | -3.49 | 68.21 | 0.0034 | ** | 2.27 | up |
| IL17A | -3.49 | 83.25 | 0.0034 | ** | 2.87 | up |
| IL1RN | -3.36 | 65.25 | 0.0047 | ** | 2.48 | up |
| IL10 | -2.87 | 76.13 | 0.0178 | * | 5.37 | up |
| IL9 | -2.33 | 95.93 | 0.0431 | * | 2.19 | up |
| IL13 | -3.55 | 108.99 | 0.0031 | ** | 1.99 | ns |
| FGF2 | -3.48 | 68.41 | 0.0034 | ** | 1.96 | ns |
| CCL7 | -2.85 | 67.05 | 0.0182 | * | 1.47 | ns |
| CCL3 | -2.80 | 64.38 | 0.0183 | * | 1.54 | ns |
| IL18 | -2.76 | 94.46 | 0.0183 | * | 1.62 | ns |
| IL8 | -2.78 | 71.96 | 0.0183 | * | 1.66 | ns |
| IL17F | -2.55 | 76.55 | 0.0303 | * | 1.89 | ns |
| IL27 | -2.53 | 143.96 | 0.0303 | * | 1.61 | ns |
| CXCL10 | -2.50 | 126.08 | 0.0304 | * | 1.62 | ns |
| MDC | 2.38 | 85.17 | 0.0405 | * | 0.84 | ns |
| TNF | -2.39 | 69.55 | 0.0405 | * | 1.48 | ns |
| PDGFA | 2.23 | 77.75 | 0.0543 | ns | 0.65 | ns |
| PDGFB | 2.15 | 99.35 | 0.0613 | ns | 0.79 | ns |
| IL5 | -2.14 | 76.07 | 0.0618 | ns | 1.55 | ns |
| CXCL1 | -2.10 | 67.27 | 0.0661 | ns | 1.66 | ns |
| TGFA | -2.09 | 62.32 | 0.0666 | ns | 1.74 | ns |
| VEGFA | 1.98 | 62.00 | 0.0813 | ns | 0.35 | ns |
| IL6 | -1.96 | 70.82 | 0.0825 | ns | 1.45 | ns |
| LTA | -1.78 | 77.99 | 0.1157 | ns | 1.36 | ns |
| CCL2 | 1.69 | 97.18 | 0.1350 | ns | 0.90 | ns |
| MCSF | -1.66 | 127.89 | 0.1371 | ns | 1.44 | ns |
| IL12A | -1.23 | 76.73 | 0.2898 | ns | 1.85 | ns |
| CXCL9 | -0.97 | 112.91 | 0.4243 | ns | 1.14 | ns |
| FLT3LG | -0.59 | 66.01 | 0.6545 | ns | 1.11 | ns |
| IL7 | 0.61 | 66.45 | 0.6545 | ns | 0.90 | ns |
| Fractalkine | -0.56 | 75.84 | 0.6580 | ns | 1.16 | ns |
| CSF3 | -0.53 | 70.63 | 0.6725 | ns | 1.21 | ns |
| sCD40L | -0.35 | 95.21 | 0.7957 | ns | 1.13 | ns |
| CCL4 | -0.17 | 87.64 | 0.9003 | ns | 1.02 | ns |
| IL22 | 0.18 | 81.74 | 0.9003 | ns | 0.89 | ns |
| EGF | -0.06 | 115.46 | 0.9510 | ns | 1.02 | ns |
| IL4 | -0.07 | 67.85 | 0.9510 | ns | 1.03 | ns |

Table S1: Differentially expressed proteins in ACPA positive sera using pairwise t-test of 45 proteins corrected by Benjamini-Hochberg. The variable *assignment* denotes a threshold of statistical significance, up: FC > 2, adjusted p-value < 0.05.

|  | **ACPA neg**  **(n = 99)** | **ACPA seroconv**  **(n = 6)** | **ACPA pos**  **(n = 30)** | **ACPA pos prog**  **(n = 12)** | **p-value** |
| --- | --- | --- | --- | --- | --- |
| **% Female (n)** | 68.7 (68) | 66.6 (4) | 76.6 (23) | 83.3 (10) | p = ns |
| **Age (IQR)** | 43.8 (22.8) | 38.0 (22.4) | 46.8 (26.9) | **29.0 (10.3)** | ACPA pos prog vs ACPA neg (p = 0.03)  ACPA pos prog vs ACPA pos (p = 0.03) |
| **BMI (IQR)** | 28 (22.8) | 35.6 (22.4) | 27.0 (26.9) | 26.5 (10.3) | p = ns |
| **CCP3 (IQR)** | **2.56 (5.9)** | 141 (30.5) | 110 (119) | 347 (166) | ACPA neg vs ACPA seroconv (p < 0.0001)  ACPA neg vs ACPA pos (p < 0.0001)  ACPA neg vs ACPA pos prog (< 0.0001) |
| **Follow-up (months, SD)** | 70.5 (178.2) | 64.8 (32.1) | 64.8 (47.8) | 81.3 (35.7) |  |

Table S2: Demographics of serum samples split by longitudinal outcome. CCP3: third generation cyclic citrullinated protein antibodies. BMI: body mass index. Analyzed by Dunn’s test and chi-square, corrected for multiple comparisons with Benjamini-Hochberg. ACPA: anti-citrullinated protein antibodies. ACPA neg: ACPA negative, ACPA seroconv: ACPA seroconversion, ACPA pos: ACPA positive, ACPA pos prog: ACPA positive progressor.

| protein | statistic | p | p.adj | p.adj.signif |
| --- | --- | --- | --- | --- |
| CCL11 | 121 | 0.13 | 0.546 | ns |
| CCL2 | 50 | 0.13 | 0.546 | ns |
| CCL3 | 91 | 0.832 | 0.979 | ns |
| CCL4 | 68 | 0.468 | 0.85673077 | ns |
| CCL7 | 65 | 0.393 | 0.80386364 | ns |
| CSF3 | 44 | 0.0737 | 0.546 | ns |
| CXCL1 | 64 | 0.369 | 0.79071429 | ns |
| CXCL10 | 54 | 0.181 | 0.546 | ns |
| CXCL9 | 76 | 0.702 | 0.979 | ns |
| EGF | 116 | 0.196 | 0.55125 | ns |
| FGF2 | 93 | 0.766 | 0.979 | ns |
| FLT3LG | 52 | 0.154 | 0.546 | ns |
| Fractalkine | 53 | 0.167 | 0.546 | ns |
| IFNA2 | 74 | 0.925 | 0.979 | ns |
| IFNG | 78 | 0.962 | 0.979 | ns |
| IL10 | 36 | 0.182 | 0.546 | ns |
| IL12A | 58 | 0.932 | 0.979 | ns |
| IL12B | 80 | 0.832 | 0.979 | ns |
| IL13 | 44 | 0.13 | 0.546 | ns |
| IL15 | 83 | 0.932 | 0.979 | ns |
| IL17A | 90 | 0.538 | 0.89666667 | ns |
| IL17F | 71 | 0.813 | 0.979 | ns |
| IL18 | 41 | 0.0539 | 0.546 | ns |
| IL1A | 79 | 0.799 | 0.979 | ns |
| IL1B | 61 | 0.304 | 0.684 | ns |
| IL1RN | 39 | 0.0432 | 0.546 | ns |
| IL2 | 67 | 0.979 | 0.979 | ns |
| IL22 | 67 | 0.712 | 0.979 | ns |
| IL25 | 38 | 0.127 | 0.546 | ns |
| IL27 | 32 | 0.0182 | 0.546 | ns |
| IL4 | 51 | 0.629 | 0.97603448 | ns |
| IL5 | 46 | 0.0898 | 0.546 | ns |
| IL6 | 76 | 0.702 | 0.979 | ns |
| IL7 | 54 | 0.181 | 0.546 | ns |
| IL8 | 84 | 0.966 | 0.979 | ns |
| IL9 | 83 | 0.932 | 0.979 | ns |
| MCSF | 69 | 0.495 | 0.85673077 | ns |
| MDC | 69 | 0.495 | 0.85673077 | ns |
| PDGFA | 105 | 0.417 | 0.81586957 | ns |
| PDGFB | 64 | 0.57 | 0.91607143 | ns |
| sCD40L | 44 | 0.13 | 0.546 | ns |
| TGFA | 60 | 0.284 | 0.67736842 | ns |
| TNF | 60 | 0.284 | 0.67736842 | ns |

Table S3: Differentially expressed proteins in paired ACPA negative samples (n = 18) for 45 proteins analysed using a paired Wilcoxon rank sum test, corrected by Benjamini-Hochberg.

| name | p-value | adjusted.p.value | Odds.ratio | score | Genes | gene.no |  |
| --- | --- | --- | --- | --- | --- | --- | --- |
| Famotidine | 1.13E-15 | 2.63E-13 | 415.8 | 14309.9 | IL10;IFNG;IL1B;IL12B;IL12A;FGF2;TNF | 7 |  |
| Diphenylpyraline | 1.24E-13 | 1.12E-11 | 80.1 | 2381.3 | IL10;IL4;IL25;IFNG;IL13;IL9;IL12B;TNF;IL2 | 9 |  |
| N-Acetylgalactosamine | 1.02E-12 | 7.56E-11 | 88.3 | 2436.8 | IL10;IL4;IFNG;IL13;LTA;IL12B;TNF;IL2 | 8 |  |
| Prednisone | 1.81E-12 | 1.23E-10 | 242.8 | 6564.7 | IL4;IFNG;IL13;IL12B;IL12A;TNF | 6 |  |
| Ciprofloxacin | 2.17E-12 | 1.41E-10 | 234.7 | 6302.9 | IL10;IL4;IL1A;IFNG;IL1B;TNF | 6 |  |
| Ibuprofen | 3.50E-12 | 2.20E-10 | 75.0 | 1979.1 | IL10;IL1A;IL1RN;IFNG;IL1B;CCL3;LTA;TNF | 8 |  |
| 9,12-Octadecadienoic Acid | 6.74E-12 | 3.93E-10 | 190.2 | 4892.6 | IL10;IL4;IFNG;IL1B;TNF;IL2 | 6 |  |
| Eicosapentaenoic acid | 7.37E-12 | 4.15E-10 | 103.6 | 2655.9 | IL10;IL4;IFNG;IL1B;IL13;TNF;IL2 | 7 |  |
| Trimethoprim | 2.59E-11 | 1.14E-09 | 57.7 | 1406.2 | IL10;IL4;IFNG;IL1B;IL13;IL12B;TNF;IL2 | 8 |  |
| Cyclosporin | 2.70E-11 | 1.16E-09 | 57.4 | 1396.1 | IL10;IL4;IFNG;IL15;IL1B;IL13;TNF;IL2 | 8 |  |
| Mevalonic Acid | 8.99E-11 | 3.19E-09 | 119.2 | 2756.2 | IL1A;IFNG;IL1B;CCL3;TNF;IL17A | 6 |  |
| Carvedilol | 8.99E-11 | 3.19E-09 | 119.2 | 2756.2 | IL10;IFNG;IL1B;IL12B;IL12A;TNF | 6 |  |
| Tacrolimus | 1.10E-10 | 3.74E-09 | 68.9 | 2756.2 | IL10;IL4;IFNG;IL15;IL13;TNF;IL2 | 7 |  |
| Retinol | 1.22E-10 | 4.07E-09 | 67.8 | 1548.5 | IL10;IL4;IFNG;IL1B;IL13;TNF;IL2 | 7 |  |
| Atorvastatin | 1.84E-10 | 5.65E-09 | 63.8 | 1430.9 | IL10;IL4;IFNG;IL1B;CCL3;TNF;IL2 | 7 |  |
| Carisoprodol | 3.39E-10 | 9.19E-09 | 58.2 | 1269.5 | IL10;IL4;IL1B;IL13;IL12B;TNF;IL2 | 7 |  |
| Acetoacetic Acid | 4.69E-10 | 1.21E-08 | 88.9 | 1909.5 | IL10;IL4;IFNG;IL13;TNF;IL2 | 6 |  |
| Maltotriose | 9.98E-10 | 2.23E-08 | 49.5 | 1026.0 | IL10;IL4;IFNG;IL1B;IL12B;TNF;IL2 | 7 |  |
| Melatonin | 1.08E-09 | 2.31E-08 | 48.9 | 1010.6 | IL10;IL4;IL1A;IFNG;IL1B;TNF;IL2 | 7 |  |
| Ethanol | 1.21E-09 | 2.46E-08 | 48.1 | 988.2 | IL10;IL4;IFNG;IL1B;IL13;TNF;IL2 | 7 |  |
| Cyclophosphamide | 1.80E-09 | 3.22E-08 | 45.3 | 912.7 | IL10;IL4;IFNG;IL15;IL13;TNF;IL2 | 7 |  |
| Rapamycin | 1.80E-09 | 3.22E-08 | 45.3 | 912.7 | IL10;IL4;IFNG;IL15;IL13;TNF;IL2 | 7 |  |
| Dexbrompheniramine | 4.89E-09 | 7.74E-08 | 58.9 | 1127.1 | IL10;IL4;IL13;CCL3;TNF;IL2 | 6 |  |
| Docosahexaenoic Acid | 6.48E-09 | 9.52E-08 | 56.1 | 1056.8 | IL10;IL4;IL1B;IL13;TNF;IL2 | 6 |  |
| Glutathione | 8.12E-09 | 1.18E-07 | 53.9 | 1003.8 | IL10;IL4;IFNG;IL1B;IL13;TNF | 6 |  |
| Deacetylchitin | 1.10E-08 | 1.50E-07 | 51.1 | 936.7 | IL10;IL4;IFNG;CCL3;TNF;IL2 | 6 |  |
| Wortmannin | 1.24E-08 | 1.66E-07 | 50.0 | 910.3 | IL1A;IFNG;IL1B;IL12B;IL12A;TNF | 6 |  |
| Methotrexate | 1.36E-08 | 1.76E-07 | 20.4 | 369.6 | IL10;IL4;IL1A;IFNG;IL1B;CCL3;TNF;IL2;IL17A | 9 |  |
| Streptozocin | 6.18E-08 | 6.19E-07 | 37.8 | 626.8 | IL10;IL4;IFNG;IL1B;TNF;IL2 | 6 |  |
| Diazepam | 6.58E-08 | 6.46E-07 | 37.4 | 617.7 | IL10;IL4;IFNG;IL13;TNF;IL2 | 6 |  |
| Alprazolam | 7.89E-08 | 7.39E-07 | 36.2 | 591.7 | IL10;IL4;IFNG;IL13;TNF;IL2 | 6 |  |
| Vitamin D3 | 8.88E-08 | 8.09E-07 | 35.4 | 575.4 | IL10;IL4;IFNG;IL13;TNF;IL2 | 6 |  |
| Epigallocatechin Gallate | 1.15E-07 | 1.03E-06 | 33.9 | 541.3 | IL10;IL4;IFNG;IL1B;TNF;IL2 | 6 |  |
| Rosiglitazone | 2.71E-07 | 2.20E-06 | 21.2 | 320.5 | IL10;IL4;IFNG;CCL3;IL12B;IL12A;TNF | 7 |  |
| Curcumin | 3.57E-07 | 2.77E-06 | 27.7 | 411.8 | IL10;IL4;IL1B;IL13;TNF;IL2 | 6 |  |
| Theophylline | 6.89E-07 | 5.04E-06 | 18.4 | 260.5 | IL4;IFNG;IL1B;IL13;IL12B;IL12A;TNF | 7 |  |
| Dronabinol | 4.80E-06 | 2.74E-05 | 17.5 | 213.7 | IL4;IL1A;IFNG;IL1B;TNF;IL2 | 6 |  |
| Troglitazone | 5.83E-06 | 3.24E-05 | 13.1 | 158.3 | IL1A;IFNG;IL1B;CCL3;LTA;TGFA;TNF | 7 | 2 |
| Bortezomib | 4.05E-05 | 0.000187852 | 9.6 | 97.1 | IL10;IL1RN;IL15;IFNA2;CCL3;TNF;IL2 | 7 | 2 |
| Genistein | 0.002188 | 0.005415152 | 5.4 | 33.1 | IL1A;IFNG;IL15;IL1B;TGFA;TNF | 6 | 3 |

Table S4: Filtered listed of drugs identified using EnrichR.
